# Multifaceted mechanisms underlying species- and cultivar-associated differential anthocyanin accumulation in fruits

**DOI:** 10.1093/hr/uhag204

**Published:** 2026-05-25

**Authors:** Ling Liang, Jiazhen Zhu, Yujie Yang, Shaojie Ai, Kui Lin-Wang, Andrew C Allan, Kunsong Chen, Changjie Xu

**Affiliations:** College of Agriculture and Biotechnology, Zhejiang University, Zijingang Campus, Hangzhou 310058, China; New Zealand Institute for Bioeconomy Science Limited, Private Bag 92169, Auckland Mail Centre, Auckland 1142, New Zealand; School of Biological Sciences, University of Auckland, Private Bag 92019, Auckland 1142, New Zealand; College of Agriculture and Biotechnology, Zhejiang University, Zijingang Campus, Hangzhou 310058, China; Cuisine Science Key Laboratory of Sichuan Province, Sichuan Tourism University, Chengdu 610100, China; New Zealand Institute for Bioeconomy Science Limited, Private Bag 92169, Auckland Mail Centre, Auckland 1142, New Zealand; New Zealand Institute for Bioeconomy Science Limited, Private Bag 92169, Auckland Mail Centre, Auckland 1142, New Zealand; School of Biological Sciences, University of Auckland, Private Bag 92019, Auckland 1142, New Zealand; College of Agriculture and Biotechnology, Zhejiang University, Zijingang Campus, Hangzhou 310058, China; Zhejiang Key Laboratory of Horticultural Crop Quality Improvement, Zhejiang University, Zijingang Campus, Hangzhou 310058, China; College of Agriculture and Biotechnology, Zhejiang University, Zijingang Campus, Hangzhou 310058, China; The State Agriculture Ministry Laboratory of Horticultural Plant Crop Growth and Development, Zhejiang University, Zijingang Campus, Hangzhou 310058, China

## Abstract

Anthocyanin is responsible for the colored hues of fruit and plays a key role in determining consumer preference and marketability. Anthocyanin accumulation exhibits remarkable diversity among fruit species and cultivars, providing invaluable genetic resources for trait improvement. This review summarizes the multifaceted mechanisms underlying the differential anthocyanin accumulation in fruit between species and cultivars. There are diverse control mechanisms governing anthocyanin accumulation, including mutations in genes involved in biosynthesis, transport, and transcriptional regulation, as well as multilayered regulation through epigenetic, post-transcriptional, and post-translational processes. The discovery of these genetic variants affecting anthocyanin accumulation, along with an understanding of the underlying molecular mechanisms, provides potential targets for fruit color modification through molecular breeding or biotechnological approaches.

## Introduction

Anthocyanins are important secondary metabolites in plants, consisting of glycosylated derivatives of anthocyanidins formed by the conjugation of one or more sugar moieties to the anthocyanidin backbone. More than 20 anthocyanidins have been identified in nature, but only six anthocyanidins are commonly found in plants: pelargonidin, cyanidin, peonidin, delphinidin, petunidin, and malvidin [1]. These anthocyanidins can form a more diverse range of anthocyanins through glycosylation at different positions with various sugar residues, including glucose, galactose, rhamnose, arabinose, and xylose [2].

Anthocyanins are water-soluble pigments that confer red, purple, and blue hues to fruits, flowers, and vegetative tissues in plants [3]. In addition to contributing to pigmentation, anthocyanins help plants attract pollinators and seed dispersers, and provide protection against stresses, such as ultraviolet radiation, drought, and cold [4, 5]. As a part of the human diet, anthocyanins play an important role in improving blood sugar balance, lowering blood lipids, and protecting against diseases, such as diabetes and cardiovascular disease due to their antioxidant activity [2, 6, 7]. With growing consumer awareness of health and nutrition, enhancing anthocyanin content in plants, especially in fresh edible fruits, has become a crucial objective in many plant breeding programs.

Anthocyanin accumulation varies among fruits due to difference in pigment type and content. For example, the differential coloration of the skin and flesh of kiwifruit (*Actinidia* spp.) is attributable to the ratio of cyanidin to delphinidin derivatives within the total anthocyanin profile [8]. However, in many fruits, differences in anthocyanin accumulation between cultivars are reflected in their anthocyanin content. Anthocyanin accumulation is regulated by genetic, developmental, and environmental factors [9, 10], with genetic background determining the ability to produce these pigments.

Existing reviews have explored the regulation of anthocyanin accumulation from various perspectives, including specific species [9, 10], types of horticultural products [11], and particular mechanisms, such as phytohormone [12, 13], nutrient deficiency [14], or environmental response [15–17]. Anthocyanin accumulation varies extensively among closely related species or cultivars, and these natural genetic variations are invaluable resources for trait improvement. In this review, after summarizing advances in knowledge of anthocyanin biosynthesis, transport, and regulation of accumulation, we focus on the molecular mechanisms underlying differential anthocyanin accumulation in fruit species and cultivars, providing a basis for screening natural variants and developing anthocyanin-enriched cultivars.

## Overview of anthocyanin biosynthesis, transport, and their regulation

### The biosynthesis and substrate competition of anthocyanins

Anthocyanins are synthesized via the phenylalanine metabolic pathway in the cytosol, which shares early-step enzymes with flavonols and proanthocyanidins (Fig. 1). These common steps involve enzymes encoded by biosynthetic genes, including phenylalanine ammonia lyase (PAL), cinnamate 4-hydroxylase, 4-coumarate coenzyme A ligase, chalcone synthase (CHS), chalcone isomerase (CHI), flavanone 3-hydroxylase (F3H), flavonoid 3′-hydroxylase, and flavonoid 3′,5′-hydroxylase. The late reaction steps are catalyzed by different enzymes: dihydroflavonol 4-reductase (DFR), anthocyanin synthase (ANS), and UDP-glucose flavonoid 3-*O*-glucosyltransferase (UFGT) for anthocyanins, flavonol synthase (FLS) for flavonols, and leucoanthocyanidin reductase (LAR) and anthocyanidin reductase (ANR) for proanthocyanidins [18]. Numerous studies have revealed that competition for common substrates can redirect metabolic flux between these pathways. For example, ANR directs flux between proanthocyanidin and anthocyanin biosynthesis, and mutations in ANR lead to variations in fruit pigmentation [19, 20]. In apple (*Malus domestica*), the dynamic competition between the biosynthetic pathways of anthocyanins and flavonols during fruit ripening is largely determined by the activities of DFR and FLS [21].

**Figure 1:**
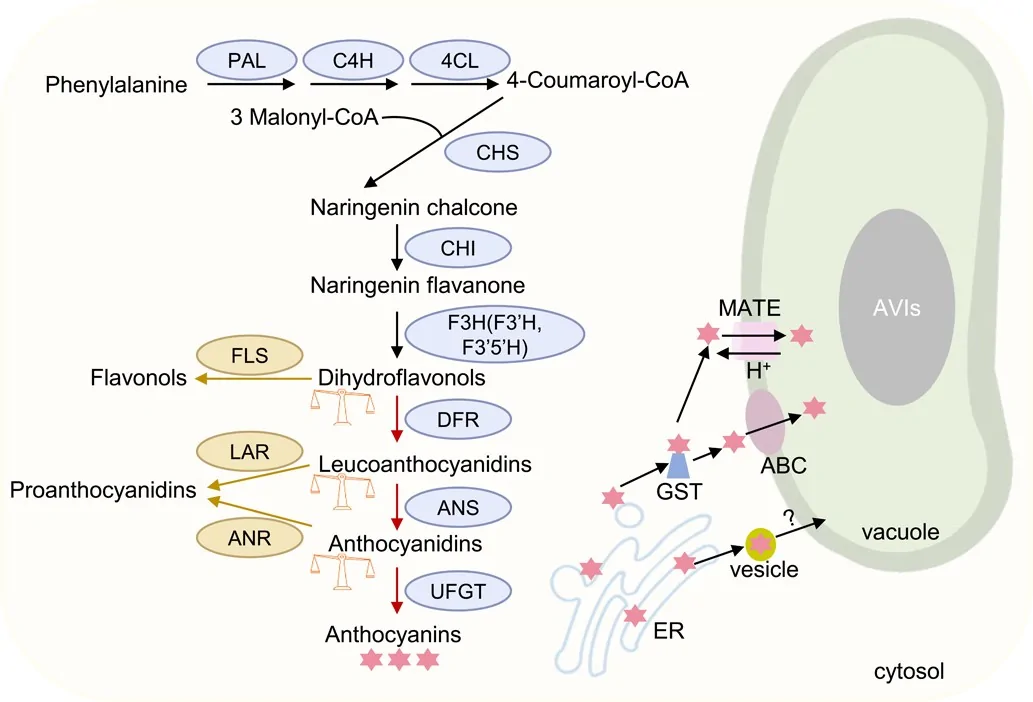
The metabolic pathway of anthocyanins. Anthocyanins are synthesized via the phenylpropanoid pathway, where metabolic flux is redirected through competition with flavonol and proanthocyanidin branches for common substrates. After biosynthesis in the cytosol, anthocyanins are transported into vacuoles via various transporters or vesicle fusion, and formed AVIs to enhance the pigment stability. ABC, ATP-binding cassette; ANR, anthocyanidin reductase; ANS, anthocyanin synthesis; CHI, chalcone isomerase; CHS, chalcone synthase; C4H, cinnamate 4-hydroxylase; DFR, dihydroflavonol 4-reductase; ER, endoplasmic reticulum; FLS, flavonol synthase; F3H, flavanone 3-hydroxylase; F3′H, flavonoid 3′-hydroxylase; GST, glutathione *S*-transferase; LAR, leucoanthocyanidin reductase; MATE, multidrug and toxic compound extrusion; PAL, phenylalanine ammonia lyase; UFGT, UDP-glucose flavonoid 3-*O*-glucosyltransferase; 4CL, 4-coumarate coenzyme A ligase.

### The transport of anthocyanins

After synthesis, anthocyanins are transported into the vacuole for storage via transporters or vesicle fusion (Fig. 1). Glutathione *S*-transferases (GSTs) have been identified as anthocyanin transporters, such as LcGST4in litchi (*Litchi chinensis*), RAP in woodland strawberry (*Fragaria vesca*), PpGST1 in peach (*Prunus persica*), and MrGST1in Chinese bayberry (*Morella rubra*) [22–25]. Many *GST* gene mutants, such as *rap* in woodland strawberry and *gst* in Japanese apricot (*Prunus mume*) [24, 26], exhibit anthocyanin-deficient phenotypes, confirming the crucial roles of *GSTs* in anthocyanin accumulation. Membrane transporters, including C-type ATP-binding cassette (ABCC) proteins and multidrug and toxic compound extrusion (MATE) proteins, are also involved in anthocyanin transport, such as ABCC1 in peach and grape (*Vitis vinifera*), ABCC8 in woodland strawberry, and MATE in grape [27–30]. Vesicle-mediated anthocyanin transport has also been observed in some plants [31, 32], with the mechanisms needing further investigation. In some plants, after being transported, anthocyanins inside the vacuole may form anthocyanic vacuolar inclusions (AVIs) by interacting with other molecules to enhance the pigment stability and color intensity [33].

### Regulation of anthocyanin accumulation

The expression of the genes encoding the biosynthesis and transport of anthocyanins are conservatively controlled at the transcriptional level by the MBW protein complex, which comprises of R2R3-MYB transcription factors (TFs), bHLH TFs, and WD40 proteins [18, 34]. In this complex, R2R3-MYB TFs act as the core regulators, while bHLH TFs and WD40 proteins act as partners [35]. Numerous R2R3-MYB anthocyanin activators and their bHLH partners have been identified, such as MdMYB1/10/110 and MdbHLH3 in apple [36–39], PpMYB10.1/2/3 and PpbHLH3 in peach [40], and MrMYB1.1*^n^* and MrbHLH1 in Chinese bayberry [41]. In contrast, several MYB TFs, including apple MdMYB16/17/111, octoploid strawberry (*Fragaria* × *ananassa*) FaMYB1, and peach PpMYB80, act as anthocyanin repressors [42–44]. They function either by interfering with the formation of MBW complexes or by directly inhibiting the transcription of *MYB* activators and structural genes [45] (Fig. 2).

**Figure 2:**
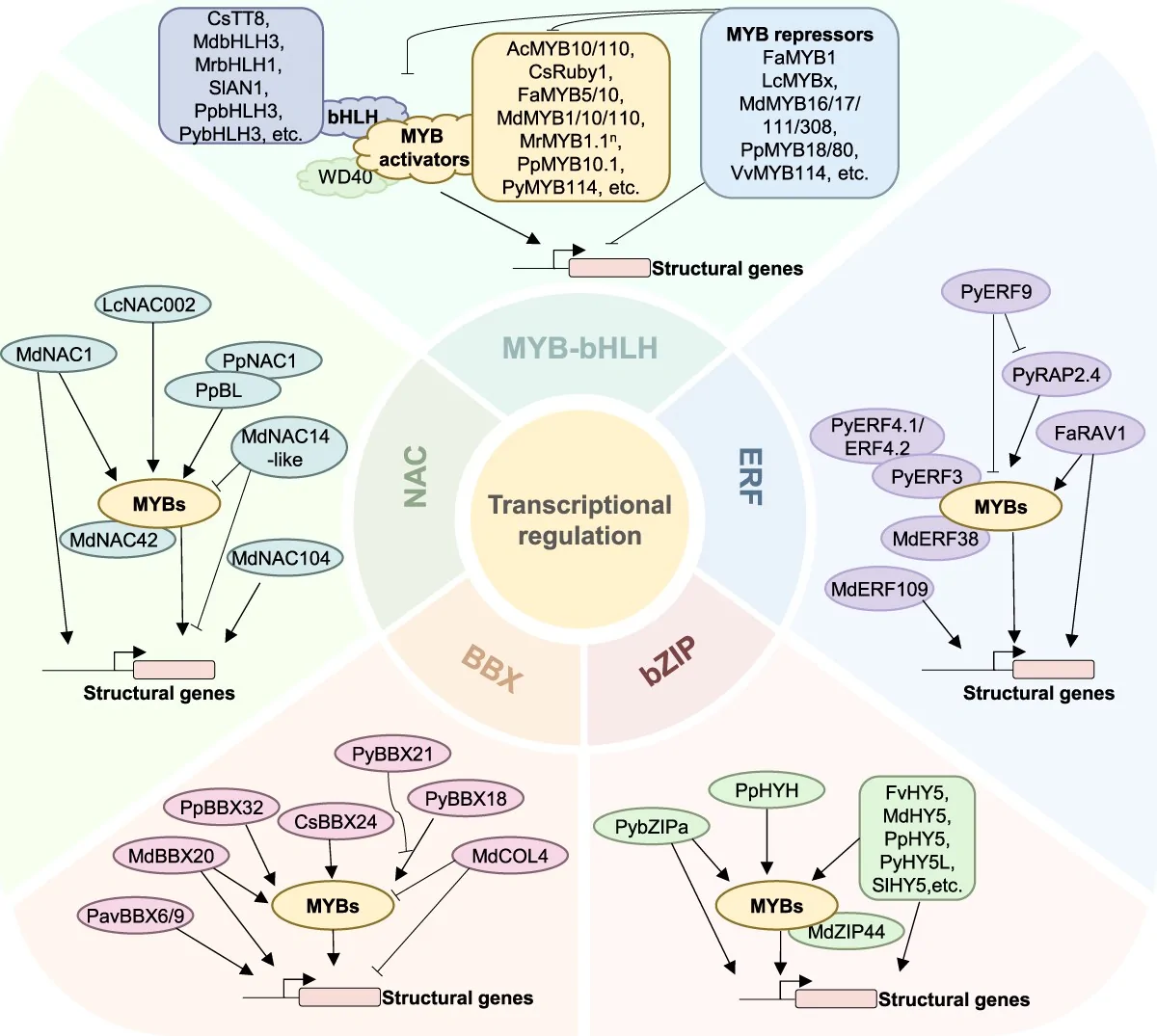
TFs involved in fruit anthocyanin accumulation. The figure summarizes TFs that regulate anthocyanin accumulation in fruits, including MYB, bHLH, NAC, ERF, BBX, and bZIP families. These TFs regulate anthocyanin accumulation by modulating the expression of key MYB TF genes or structural genes. Arrows and blunt-ended lines indicate positive and negative regulation of target gene expression, respectively. Ac, *Actinidia chinensis*; Cs, *Citrus sinensis*; Fa, *Fragaria* × *ananassa*; Fv, *Fragaria vesca*; Lc, *Litchi chinensis*; Md, *Malus domestica*; Mr, *Morella rubra*; Pav, *Prunus avium*; Pp, *Prunus persica*; Py, *Pyrus pyrifolia*; Sl, *Solanum lycopersicum*; Vv, *Vitis vinifera*.

Other TF families are also involved in anthocyanin accumulation, such as NAC, ERF, BBX, and bZIP families, either by directly targeting anthocyanin biosynthesis-related genes or by forming transcriptional cascades and protein complexes between other TFs (Fig. 2). For example, bZIP family TFs HY5 and HY5-like mediate anthocyanin accumulation via activating anthocyanin-related *MYBs* and structural genes in many fruits [46, 47]. MdNAC104 promotes anthocyanin accumulation in apple by upregulating the expression of anthocyanin biosynthetic genes [48]. Pear (*Pyrus pyrifolia*) BBX18 and BBX21 antagonistically regulate anthocyanin biosynthesis by competitive association with HY5, blood orange (*Citrus sinensis*) BBX24 regulates anthocyanin biosynthesis by activating the MYB gene *Ruby1* transcription [49, 50]. PyERF4.1 and PyERF4.2 interact with PyERF3, thereby modulating the stability of the PyERF3–PyMYB114–PybHLH3 complex and repressing anthocyanin accumulation in pear [51].

In addition, research indicates the involvement of epigenetic, post-transcriptional and post-translational regulation in fine-tuning anthocyanin accumulation (Fig. 3), including DNA methylation, microRNA (miRNA) regulation, alternative splicing (AS), protein modification, and more. These will be discussed in the subsequent sections on species/cultivar-associated differential anthocyanin accumulation in fruits.

**Figure 3:**
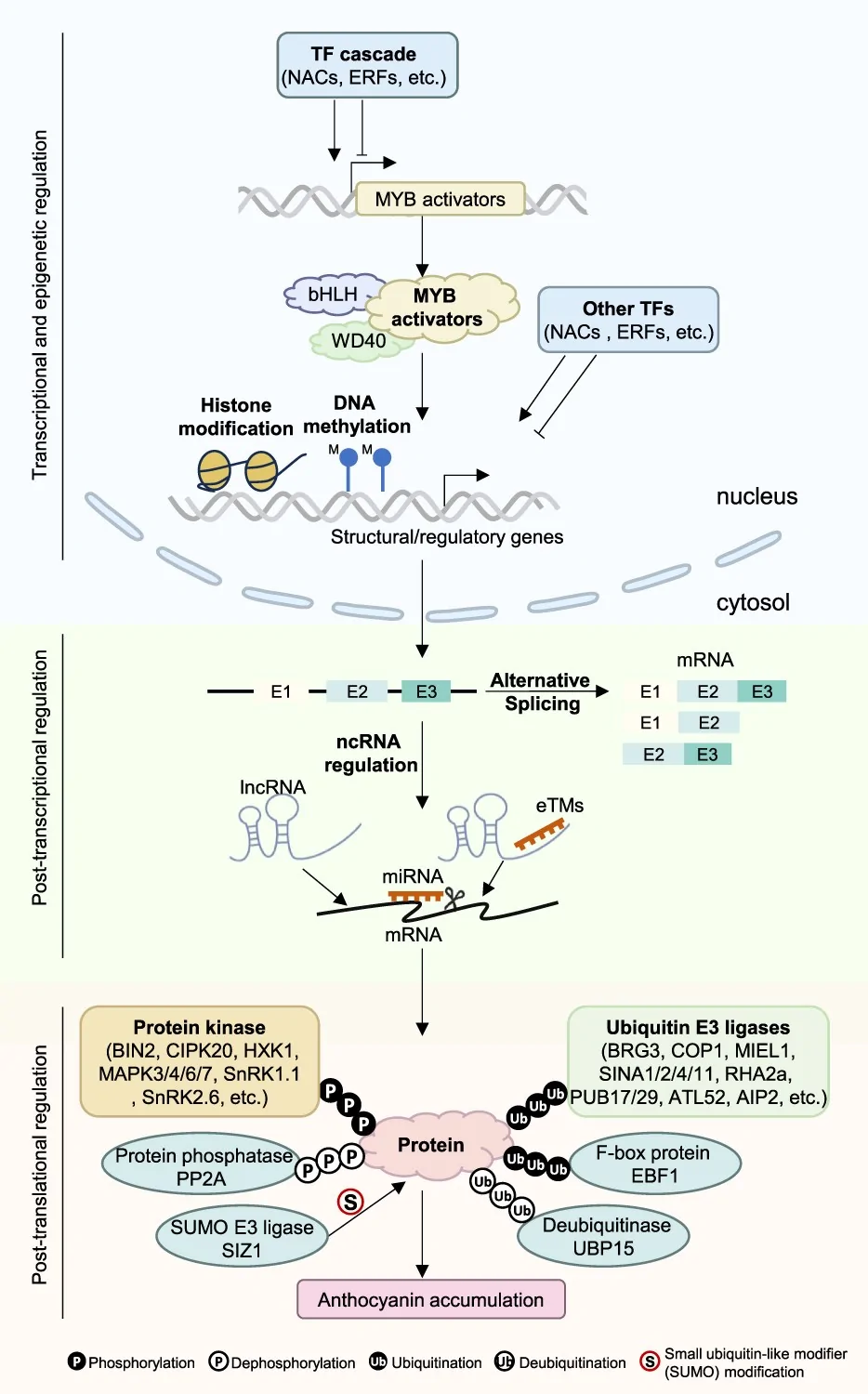
Regulatory mechanisms of anthocyanin accumulation. This schematic diagram illustrates the multilayered regulatory network controlling anthocyanin accumulation, including transcriptional, epigenetic, post-transcriptional, and post-translational regulation. Arrows and blunt-ended lines indicate activation and repression, respectively. E1/2/3, exon 1/2/3; eTMs, endogenous target mimics; lncRNA, long noncoding RNA; M, DNA methylation; miRNA, microRNA; ncRNA, noncoding RNA; TF, transcription factor.

## Mutations in gene coding regions affecting protein function

### Mutations of anthocyanin biosynthetic and transport genes

Genetic studies have revealed aberrant anthocyanin accumulation in some cultivars due to mutations in the coding regions of anthocyanin biosynthetic/transport genes. Amino acid mutations in the F3H and PAL2 proteins reduce catalytic activity, resulting in pink and light-red woodland strawberry fruits, respectively [52, 53]. A 37.2-kb chromosome translocation disrupted the coding region of *PgANS*, causing anthocyanin deficiency in white pomegranate (*Punica granatum*) [20]. Regarding anthocyanin transport genes, the premature terminations in the *GST* coding regions caused by base substitutions result in anthocyanin deficiency in woodland strawberry and Japanese apricot [24, 26]. These mutant genes have been confirmed as key genes controlling color traits by gene overexpression or gene editing technologies [20, 24, 26, 52, 53]. The red-peel phenotype of some walnuts (*Juglans regia*) is proposed to be associated with several single nucleotide polymorphisms (SNPs) in the coding region of *JrUFGT5* [54], but further validation was not available.

### Mutations of transcription factor genes

Some natural fruit anthocyanin variations converge on functional changes in TF genes. Loss-of-function mutations in anthocyanin activators often result in naturally anthocyanin-deficient variants. Compared to red strawberry fruit, yellow and white fruits lack anthocyanin accumulation due to different types of *MYB10* loss-of-function mutations. An SNP in *FvMYB10* has been identified as the causal determinant of yellow fruit in woodland strawberry [55]. In white-fruited woodland strawberry, a gypsy transposon has inserted in the third exon of *FvMYB10* to disrupt anthocyanin biosynthesis [56]. In white-fruited octoploid strawberry, an 8-bp insertion and an AG-insertion in the *FaMYB10* coding sequence result in truncated proteins and anthocyanin deficiency [57, 58]. In white grape, Walker *et al.* [59] identified two mutations in the *VvMYBA2* coding region, including an amino acid substitution and a frameshift mutation. In white Chinese bayberry, a 1-bp deletion in *MrMYB1.1* coding region caused a loss of function [41, 60]. The pink-fruited blueberry (*Vaccinium corymbosum*) mutant is associated with the mutation of *VcMYB1* [61]. These genes have been functionally validated as the determinants of color variations [59–64]. In sweet cherry (*Prunus avium*), *PavMYB10.1a* and *PavMYB10.1b* with a 1-bp deletion were identified in red-skinned and blush-skinned fruits, respectively, while an ~90-kb deletion encompassed *PavMYB10.1* produced a nonfunctional allele, named *PavMYB10.1_Del_* or *PavMYB10.1c*, and resulted in yellow-skinned fruit. The marker developed on the sequence polymorphism of the three *PavMYB10.1* alleles provides a useful molecular tool for the selection of fruit color [62, 63]. In ‘Wushancuili’ plum (*Prunus salicina*), anthocyanin deficiency in peel is related to the loss-of-function of four *MYB10* genes [64]. Hawthorn (*Crataegus pinnatifida*) fruits are generally red-skinned, but a few cultivars are yellow, which is related to a base substitution in *CpMYB114* [65].

In contrast, natural variants with increased anthocyanin accumulation can result from loss-of-function mutations of repressors and gain-of-function in activators. Most cultivated tomatoes (*Solanum lycopersicum*) lack anthocyanin accumulation in fruit, but some wild species show purple coloration, which is associated with a 4-bp insertion in the coding region of the R3-MYB repressor *SlMYBATV* [66]. In pear, a 14-bp deletion in the *PyBBX24* coding region results in premature translation termination and loss of its function in inhibiting anthocyanin biosynthesis, leading to red peel coloration [67, 68]. In red-skinned longan (*Dimocarpus longan*), a gain-of-function mutation of three SNPs in the *DlMYB113* coding sequence promotes anthocyanin accumulation, and this variation has been utilized to develop molecular markers for the early screening of longan hybrids with desired fruit color [69].

In addition, some anthocyanin-related *MYBs* undergo genome and tandem duplication, leading to functional divergence and anthocyanin variation. In Chinese bayberry, an *MYB* gene cluster comprising *MrMYB1.1*, *MrMYB1.2*, *MrMYB1.3*, and *MrMYB2* has been identified. Among these, only *MrMYB1.1^n^* and *MrMYB1.3* enhance anthocyanin accumulation, whereas *MrMYB1.1^d^* (the allele of *MrMYB1.1^n^*), *MrMYB1.2*, and *MrMYB2* lack this function. Their diverse functions, expression patterns, and allele-specificity contribute to the differential anthocyanin accumulation among cultivars, resulting in colors ranging from purple, red, and white. The established cleaved amplified polymorphic sequence markers targeting *MrMYB1.1^n^*/*MrMYB1.1^d^* exhibit the ability to quickly distinguish cultivars with different colored ripe fruits [41]. In apple, two *MYB* gene clusters regulating anthocyanin accumulation have been identified: *MdMYB10* (and its allele *MdMYB1*) and *MdMYB90-*like on chromosome 9, and *MdMYB110a* and *MdMYB110b* on chromosome 17, although MdMYB110b is nonfunctional due to protein truncation. *MdMYB1* and *MdMYB90-*like regulate fruit peel coloration, while *MdMYB10* and *MdMYB110a* control the formation of type I and type II red-fleshed traits, respectively [36–38, 70].

## Changes affecting gene expression

### Mutations in promoter regions of anthocyanin biosynthetic and transcription factor genes

Unlike mutations that occur in coding regions, which affect the function of the encoded protein, mutations in promoter regions affect the expression of genes and can therefore also lead to altered anthocyanin accumulation. There are several reports on biosynthetic genes and numerous on TF genes.

In non-purple eggplant (*Solanum melongena*), the lack of anthocyanin is due to a SNP at the MYB recognition site of the *DFR* promoter, which leads to decreased expression of *DFR* [71]. In purple kiwifruit (*Actinidia arguta*), a 29-bp insertion in the *AaLDOX* promoter affects promoter activity, and is associated with coloration [72]. In pomegranate, a repeat expansion in the *PgANR* promoter results in reduced expression, limiting the conversion of anthocyanins to proanthocyanidins and leading to the black peel phenotype [20].

For TF genes, transposon insertions in promoter regions often alter gene expression, leading to anthocyanin-enriched or anthocyanin-deficient variants. In peach, an NAC TF, named *PpBL*, was identified as the key gene for the blood-fleshed trait [73]. Further research revealed that the specific high expression in blood-flesh peach is due to a 6688-bp long terminal repeat (LTR) retrotransposon insertion in the promoter of *PpBL*, which attenuates the repressive effect on TF PpWRYK70 [74]. In red-fleshed plum, an LTR retrotransposon in the promoter of *PsMYB10.2* triggers anthocyanin biosynthesis [75]. Blood orange gains anthocyanin accumulation ability due to a retrotransposon insertion in the *Ruby1* promoter [76]. In red-fleshed octoploid strawberry, a CACTA-like transposon in the *FaMYB10-2* promoter increases its expression and anthocyanin accumulation, which can be targeted for marker development for the selection of fruit color [56]. In red-skinned apple, a gypsy-like LTR retrotransposon named redTE in the *MdMYB1* promoter determines coloration by enhancing *MdMYB1* expression, and the redTE-based specific marker is a valuable tool for preselection of hybrid seedlings with objective skin color [77]. A non-LTR retrotransposon in the MYB TF *CaAn2* promoter activates its expression, causing anthocyanin accumulation in purple-pigmented pepper (*Capsicum annuum*) [78]. In white grape, a retrotransposon *Gret1* insertion in *VvMYBA1* promoter disrupts its transcription and blocks anthocyanin accumulation [79].

Previous studies have shown that other types of mutations in promoters, such as fragment insertions, SNPs, and InDels, can also regulate the expression of TF genes and cause differences in anthocyanin accumulation. The presence of multiple repeats of a promoter segment causes *MdMYB10* autoregulation, controlling the type I red-fleshed apple phenotype, while a 163-bp InDel in the *MdWRKY10* promoter confers various degrees of red pigmentation [80, 81]. Most cultivated citrus fruits do not accumulate anthocyanin due to mutations in the TATA-box and CAAT-box regions of the *Ruby1* promoter, which repress its transcription [82]. However, purple pummelo (*Citrus grandis*) without these mutations accumulates anthocyanin in the peel [82]. Promoter sequence variations, including eight deletions and two insertions, decrease the promoter activity of *FnMYB10*, resulting in the white Nilgiri strawberry (*Fragaria nilgerrensis*) phenotype [83].

### Gene expression changes related to DNA methylation

DNA methylation can change gene expression via adding a methyl group to cytosine bases without altering the sequence, and thereby regulate anthocyanin biosynthesis by directly affecting the expression of structural genes or TF genes, as observed in a number of fruits [84]. Methylation of the *MdMYB1* promoter is responsible for the coloration of different blushed sports in apple, with lower methylation levels leading to higher anthocyanin content [85, 86]. In a ‘Fuji’ apple mutant, the lower level of methylation in the *MdMYB90-like* promoter explains increased expression and enhanced red pigmentation accumulation [70]. In yellow- and green-skinned mutant apples, anthocyanin deficiency is associated with *MdMYB10* hypermethylation [87, 88]. The differences in anthocyanin accumulation patterns between red and green stripes are also due to differential methylation levels in the *MdMYB10* promoter [89]. In pear (*Pyrus* spp.), the high methylation level of the *MYB10* promoter inhibits anthocyanin accumulation in the green-skinned sport of ‘Max Red Bartlett’ [90], whereas demethylation of the promoter of *MYB10* contributes to the red-striped coloration in bud sport ‘Red Zaosu’ [91]. DNA methylation variation in the *VvMYBA1* promoter determines color differences between white- and red-fleshed grape [92], as well as the coloration of the ‘Manicure Finger’ grape with a red-purple top and yellow-green base [93]. The contrasting anthocyanin levels in purple- and green-skinned fig (*Ficus carica*) are associated with the DNA methylation level of the *FcUFGT* promoter [94].

### Differential expression between cultivars with unrevealed mechanisms

Certain fruit cultivars gain or lose anthocyanin due to enhanced expression of activators or repressors, yet the underlying regulatory mechanisms controlling their expression remains to be discovered. In purple kiwifruit, the high expression of the activator *MYB110* and the low expression of the repressor *AaBEE1* (a gene coding a bHLH TF) contribute to anthocyanin accumulation in both flesh and peel [8, 72]. In some red-centered kiwifruit species, *AcMYB10* and *AcMYB123* are responsible for anthocyanin accumulation [95, 96]. The different colors of mango cultivars, ranging from purple, red, yellow, and green, are associated with differential expression of *MiMYB33* [97]. The expression of *PavMYB.C2* determines color variation between the yellow- and red-fruited sweet cherry [98]. In red-fleshed pear, *PcERF5* is highly expressed and identified as an anthocyanin activator [99]. In contrast, pear *MADS18*, *ANR*, and *LAR* are associated with the anthocyanin deficiency in green bud sports [100, 101], while in yellowed-skinned peach and apple, the lack of anthocyanin accumulation is due to the high expression of MYB repressors *PpMYB80* and *MdMYB28*, respectively [43, 102]. The repressor *FcMYB1* is highly expressed in white strawberry (*Fragaria chiloensis*) fruit and determines the discolored phenotype [103].

**Table 1:** ncRNAs involved in fruit anthocyanin accumulation.

**ncRNAs**	**Regulatory module**	**Species**	**Function**	**References**
miRNAs	miR21-*CHI*	*V. ashei*	Repression	[111]
	miR156-*SPL*	*A. chinensis*	Activation	[95]
		*H. rhamnoides*	Activation	[113]
		*L. chinensis*	Activation	[112]
		*M. domestica*	Repression	[109]
		*P. granatum*	Activation	[119]
		*P. pyrifolia*	Activation	[107]
		*V. corymbosum*	Activation	[108]
	miR157-*SPL*	*P. granatum*	Activation	[119]
		*S. melongena*	Repression	[120]
	miR160-*ARF16*	*A. chinensis*	Activation	[95]
	miR164-*NAC22/100*	*P. persica*	Repression	[110]
	miR167-*F3H*	*V. ashei*	Repression	[111]
	miR171-*SCL6*	*A. chinensis*	Activation	[95]
	miR394-*F-box1*	*A. chinensis*	Activation	[95]
	miR395-*APS1*	*M. domestica*	Repression	[121]
	miR397-*LAC11a*	*F. vesca*	Repression	[114]
	miR408-*BBP*	*M. domestica*	Activation	[122]
	miR477-*ABCB19AS*	*Morus atropurpurea*	Activation	[117]
	miR828-*MYB*	*H. rhamnoides*	Repression	[113]
		*S. lycopersicum*	Repression	[123]
	miR828-*TAS4*	*A. chinensis*	Repression	[95]
		*V. vinifera*	Activation	[105]
		*M. domestica*	Repression	[104, 124]
	miR858-*MYB*	*A. arguta*	Repression	[125]
		*M. domestica*	Activation	[106]
		*S. lycopersicum*	Repression	[123]
		*V. vinifera*	Activation	[105]
		*V. ashei*	Repression	[111]
	miR5072-*ANR*	*M. domestica*	Activation	[126]
	miR5290- *MADS1*	*Fragaria × ananassa*	Activation	[127]
	miR7125-*CCR*	*M. domestica*	Activation	[128]
	NEW41-*CHI*	*L. chinensis*	Repression	[112]
siRNAs	TAS4-siRNA-*MYB*	*V. vinifera*	Activation	[105]
		*M. domestica*	Repression	[124]
		*A. chinensis*	Repression	[95]
	TAS4-siR81(−)-*bHLH3*	*M. domestica*	Repression	[104]
	vsiR693-*PIF1*	*M. domestica*	Repression	[118]
lncRNAs	LNC1-miR156a	*H. rhamnoides*	Repression	[113]
	LNC2-miR828a	*H. rhamnoides*	Activation	[113]
	MLNC3.2/4.6-miR156	*M. domestica*	Activation	[109]
	FRILAIR-miR397	*F. vesca*	Activation	[114]
	MdLNC499-*MdERF109*	*M. domestica*	Activation	[116]
	MdLNC610-*MdACO1*	*M. domestica*	Activation	[115]
	Mul-ABCB19AS-*Mul-ABCB19*	*M. atropurpurea*	Repression	[117]

## Mutations that affect post-transcriptional and post-translational regulation of anthocyanin accumulation

### Differential expression of noncoding RNA

miRNAs are a class of endogenous noncoding small RNAs of 19–24 nucleotides (nt) which are extensively involved in the regulation of anthocyanin accumulation (Table 1). The regulatory functions of several miRNAs are conserved across fruit species. For example, miR828 cleavage of the noncoding *TRANSACTING SiRNA GENE 4* (*TAS4*) transcript generates a phased small interfering RNA (siRNA) that targets anthocyanin-related *MYBs* in apple and kiwifruit [95, 104]. miR858 targets anthocyanin-related *MYB* activators or repressors in apple and grape, thereby affecting fruit anthocyanin accumulation [105, 106]. The miR156-*SPL* regulatory module regulating anthocyanin accumulation is conserved in apple, highbush blueberry, and pear, as SPL TFs interfere with the formation of the MBW complex [107–109]. In contrast, some miRNAs exhibit species-specific roles in anthocyanin accumulation regulation. In peach, miR164 targets *PpNAC22* and *PpNAC100* to regulate tissue-specific anthocyanin accumulation [110]. miR167 and miR21 directly target anthocyanin biosynthetic genes *F3H* and *CHI* in rabbiteye blueberry (*Vaccinium ashei*), respectively [111]. Moreover, a novel miRNA NEW41 is identified as a regulator of anthocyanin accumulation in litchi [112]. Recently, differential expression of miRNAs was found to account for differences in anthocyanin accumulation among other plant cultivars. For example, miR828 and miR858 regulate *VvMYB114* to confer grape peel color, which ranges from green, red, and purple [105]. In kiwifruit selections with a red inner flesh, miR156, miR160, miR171, and miR394 are differentially expressed between the inner and outer flesh, and the products of their target genes can interact with the MYB10 protein, leading to tissue-specific anthocyanin accumulation [95].

In addition to miRNA, siRNA and long noncoding RNAs (lncRNAs) also participate in the regulation of anthocyanin accumulation (Table 1). The lncRNAs have been found to act as endogenous target mimics (eTMs) of miRNAs to block miRNA-mediated cleavage of target genes, thereby regulating anthocyanin accumulation in many fruits. For example, in sea buckthorn (*Hippophae rhamnoides*) fruit, LNC1 and LNC2 act as eTMs of miR156a and miR828a to regulate the expression of *SPL9* and *MYB114*, respectively [113]. *FRILAIR* positively regulates woodland strawberry coloration by acting as a noncanonical target mimic of miR397 [114]. In apple, the mechanisms of lncRNAs-mediated anthocyanin accumulation are diverse. MLNC3.2 and MLNC4.6 act as eTMs for miR156a, whereas *MdLNC610* and *MdLNC499* promote anthocyanin accumulation by activating the expression of *MdACO1* and *MdERF109* [115, 116]. In mulberry (*Morus alba*), lncRNA *Mul-ABCB19AS* can suppress *Mul-ABCB19* expression, inhibiting anthocyanin accumulation [117]. A viroid-derived siRNA vsiR693 inhibits apple anthocyanin biosynthesis via targeting bHLH TF gene *MdPIF1* [118]. However, whether siRNAs and lncRNAs contribute to species- and cultivar-associated anthocyanin accumulation needs further research.

### Alternative splicing

AS is a process that generates multiple mature mRNAs from a single gene, and is widely involved in biological processes, including regulation of anthocyanin accumulation [10, 129]. In tomato, most domesticated cultivars lack anthocyanin in their fruit due to AS of the MYB TF gene *SlAN2-*like which produces a truncated nonfunctional protein [130]. In non-purple eggplant mutant, an SNP at the 5′ splicing site of the second intron of *DFR* results in an AS variant responsible for the anthocyanin deficiency [71]. In purple striped pepper mutant, the functional AS of the MYB TF gene *CaAN3* affects fruit anthocyanin biosynthesis [131].

### Post-translational regulation

There is increasing evidence for the involvement of post-translational protein modifications in anthocyanin accumulation, including protein phosphorylation, ubiquitination and small ubiquitin-like modifier (SUMO) modification (SUMOylation).

Among post-translational modifications, protein phosphorylation is particularly prevalent and is regulated by protein kinases and protein phosphatases. In strawberry, the mitogen-activated protein kinase MAPK3 negatively regulates anthocyanin accumulation by phosphorylating MYB10 and CHS1 [132], while the SNF1-related kinase SnRK2.6 acts as a repressor by regulating MAPK3 or directly phosphorylating bHLH3 [133]. In apple, several protein kinases have been identified to regulate anthocyanin accumulation, including MAPK4, MAPK6, calcineurin B-like protein-interacting protein kinase CIPK20 and SnRK1.1 [134–137]. In pear, the HB40 (an HD-Zip family TF)-*MYB123-*like module mediates anthocyanin accumulation, which is regulated by the protein phosphatase PP2A [138]. Examples of differential protein phosphorylation involved in anthocyanin accumulation among cultivars are emerging. For example, it has been observed that ‘Nanhong’ pear, a red mutant of ‘Nanguo’, exhibits differential anthocyanin accumulation associated with the expression level of a protein kinase gene that mediates the phosphorylation of anthocyanin-related genes [139].

Protein ubiquitination affects anthocyanin accumulation by degrading proteins, related to this phenotype, via the 26S proteasome. Ubiquitin E3 ligases are key components of this proteasome and have been identified in some fruits. For example, E3 ligase COP1 ubiquitinates various proteins and triggers their degradation, including bZIP, MYB, bHLH, and NAC family members, to negatively regulate anthocyanin accumulation [140–142]. In apple, many E3 ligases are involved in regulation of anthocyanin accumulation, such as MdMIEL1, MdSINA1/2/4/11, MdRHA2a, and MdPUB29 [137, 143–148]. The deubiquitinase MdUBP15 and the E3 ligase MdPUB17 coordinate the TF MdGLK1 (GOLDEN2-LIKE 1) stability in apple anthocyanin deposition [149]. In pear, the E3 ligase PyATL52 mediates the degradation of HEAT SHOCK FACTOR B2A (PyHsfB2a) to affect anthocyanin accumulation [47]. Recently, it has been reported that protein ubiquitination is involved in the cultivar-specific anthocyanin accumulation. In white-fleshed pears, the HD-ZIP TF PyHAT5 interferes with the formation of the PyMYB10–PybHLH3 complex and inhibits anthocyanin accumulation. However, in red-fleshed pears, the E3 ubiquitin ligase PyAIP2 targets PyHAT5 for ubiquitination and degradation, thereby relieving the inhibitory effect of PyHAT5 on anthocyanin biosynthesis, ultimately resulting in red-flesh trait [150].

In addition, increasing research reveals the interaction between ubiquitination and phosphorylation in anthocyanin accumulation regulation in apple. The protein kinase MdMAPK7 antagonistically regulates the E3 ubiquitin ligase MdBRG3-mediated degradation of MdLBD36 (a protein with LATERAL ORGAN BOUNDARIES domain), thereby maintaining the stability of MdLBD36 and promoting anthocyanin biosynthesis [151]. The F-box protein MdEBF1 and MdMAPK7 antagonistically regulate DELLA protein MdRGL2a homeostasis to promote anthocyanin biosynthesis [152]. The E3 ubiquitin ligase MdSINA1 and the protein kinase MdBIN2 cooperatively regulate MdPHR1 (a TF that serves as the master regulator of the inorganic phosphate starvation response) in anthocyanin biosynthesis [153]. However, these studies all focus on anthocyanin accumulation induced by environmental factors or hormonal signals; it remains unclear whether protein ubiquitination and phosphorylation co-regulate species- and cultivar-associated differential anthocyanin accumulation.

SUMO can hinder the combination of ubiquitin and target proteins, increasing the stability of target proteins. For example, in apple, the SUMO E3 ligase MdSIZ1 SUMOylates MdMYB1 and promotes anthocyanin accumulation under low temperature [154]. Further research found that MdMYB2 can directly bind to the *MdSIZ1* promoter and activate its transcription, forming MdMYB2–MdSIZ1–MdMYB1 regulatory cascade [155]. However, such studies have not been extended to cultivar difference in anthocyanin accumulation.

**Figure 4:**
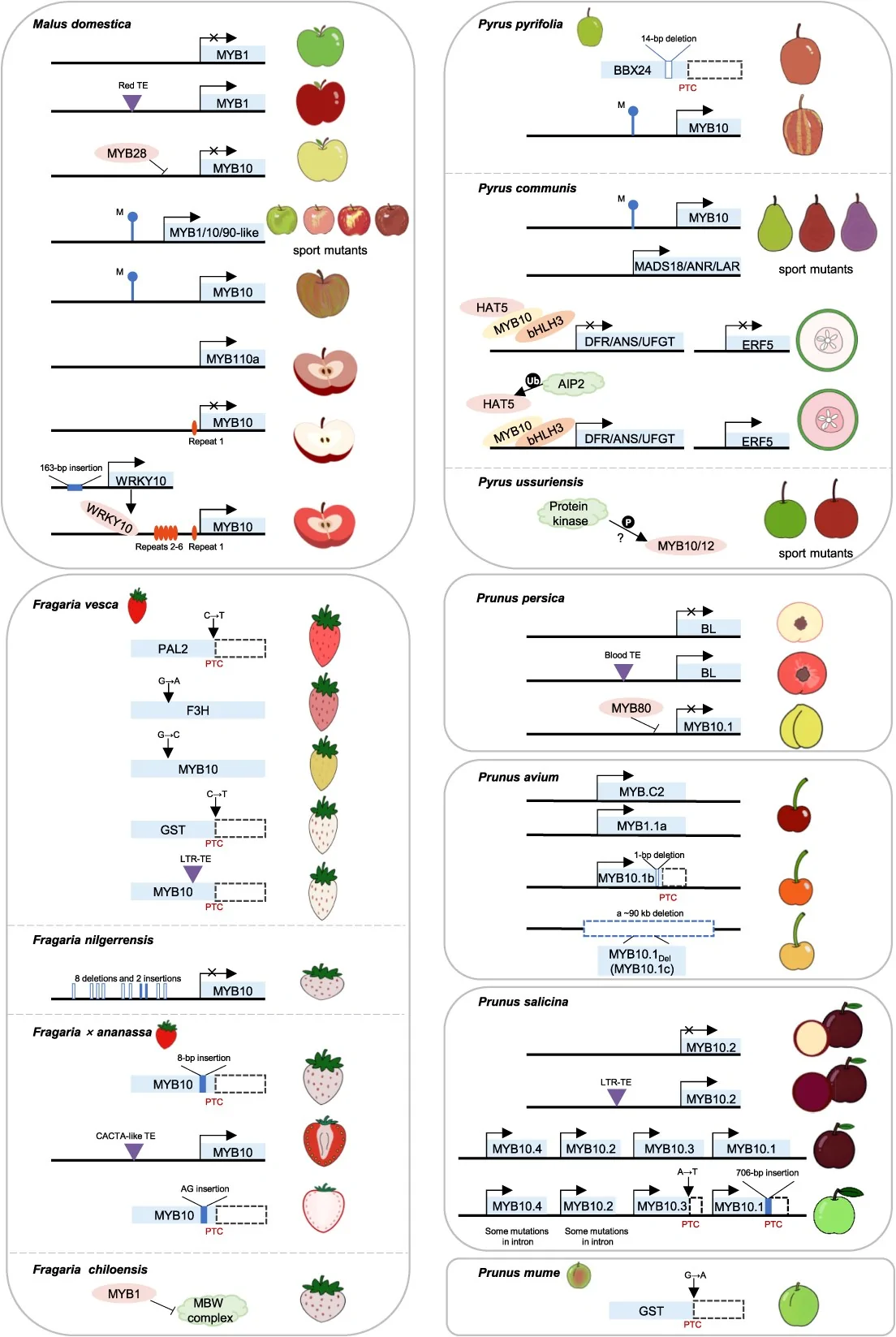
Anthocyanin variants in Rosaceae fruit. This figure illustrates the molecular mechanisms underlying anthocyanin variants in various fruits, including mutations in genes involved in biosynthesis, transport, and transcriptional regulation, as well as multilayered regulation through epigenetic, post-transcriptional, and post-translational processes. Arrows and blunt-ended lines indicate positive and negative regulation on target gene, respectively. M, DNA methylation; P, phosphorylation; PTC, premature termination codon; Ub, ubiquitination. The triangles represent transposon insertion.

## Conclusions and prospects

Anthocyanin plays an important role in the appearance and nutritional value of fruits. The genetic background of cultivar selection determines the ability of anthocyanin to accumulate in fruits, and this review summarized the natural anthocyanin variants resulting from specific genetic mutations. The underlying mechanisms are diverse, involving mutations in anthocyanin pathway genes, as well as transcriptional, epigenetic, post-transcriptional, and post-translational regulation (see Fig. 4 for examples present in Rosaceae fruit). Certain anthocyanin variants, such as white strawberry and red-skinned pear, exhibit higher commercial value compared with the ordinary cultivars. The discovery of these variants, along with an understanding of the underlying molecular mechanisms associated with phenotype, can provide potential targets for fruit color modification through molecular breeding. However, most perennial crops have a long juvenile phase that prolongs the genetic improvement process and increases input costs. Marker-assisted selection and gene editing technologies have emerged as crucial strategies for increasing breeding efficiency [156]. Many genetic variations provide valuable resources for developing molecular markers, such as the variations of apple *MYB1*, longan *MYB113*, and Chinese bayberry *MrMYB1.1* [41, 69, 77]. Clustered Regularly Interspaced Short Palindromic Repeats/CRISPR-associated protein 9 (CRISPR/Cas9)-mediated gene editing targets the regulators of anthocyanin accumulation, successfully altering the fruit color [20, 157]. However, it should be recognized that for certain commercially important fruit species, the molecular mechanisms underlying intraspecific variation of anthocyanin levels or composition remain to be explored, such as avocado, olive, and European plum. Advances in modern genome sequencing can facilitate the understanding of diversity in anthocyanin accumulation within a species or between species. For example, the whole-genome sequencing results revealed that coloration differences among pomegranate cultivars are due to *ANS* and *ANR* mutations [20]. The genome-wide association studies indicate that a large inversion (~12.4 Mb) strongly associates with the fruit color in eggplant [158].

In summary, anthocyanins are synthesized via a conserved phenylalanine metabolic pathway, which is regulated at the transcriptional, epigenetic, post-transcriptional, and post-translational levels (Figs 1 and 3). Some of these regulatory mechanisms are conserved among species, such as MBW complex, miR156-*SPL*, and miR858-*MYB* modules. However, the mechanisms underlying most natural anthocyanin variants are species-specific (Fig. 4). For example, the formation of blood-flesh peach and blood orange traits is due to the *BL* and *Ruby* mutation, respectively [74, 76]. The combination of conserved modules and species-specific adaptations reveals the evolutionary flexibility of anthocyanin regulatory networks. In addition, some mysteries regarding the regulation of anthocyanin accumulation at post-transcriptional and post-translational levels have been revealed through development- or environment-based studies. For example, miR7125 regulates light-induced anthocyanin accumulation in apple, while ubiquitination of HsfB2a mediates anthocyanin accumulation under high temperature in pear [47, 128]. Whether these mechanisms are involved in cultivar-dependent anthocyanin accumulation remains to be studied. Expanding multi-omics approaches, including comparative genomics as well as whole-transcriptome, methylome and proteome analyses, can be conducted to investigate the regulatory mechanisms of cultivar-specific anthocyanin accumulation in more fruit species.

The study of species- and cultivar-associated differential anthocyanin accumulation in fruits has made significant progress, but several key questions remain unanswered:

1) Crosstalk between different regulatory layers: this review summarizes the regulation of anthocyanin accumulation on transcriptional, epigenetic, post-transcriptional, and post-translational levels. However, the crosstalk between these layers remains unclear. For example, how do epigenetic modifications influence the binding of TFs to target gene promoters?
2) Upstream regulators of key TFs: multiple layers of TFs interact with the MBW complex, including NACs, BBXs, and ZATs, and their differential expression has been observed among cultivars. For example, the repressor *PpMYB80* accounts for skin color variation in peach, but its upstream regulators remain unidentified [43]. Future work can focus on revealing transcriptional regulatory networks using co-expression network analysis and genome-wide identification.
3) The potential pleiotropic effects of anthocyanin regulators: the MYB TF MdCPCL simultaneously activates ascorbic acid and anthocyanin accumulation in red-fleshed apple, CsBBX24 regulates both carotenoid and anthocyanin biosynthesis in citrus [50, 159]. PpBL controls the peach blood-flesh trait formation, but it reduces aroma and increases sourness [73, 160, 161]. Therefore, when utilizing such genes as targets for anthocyanin-enriched fruit breeding, it is essential to consider the integration and balance of multiple quality traits.
4) The interaction between environmental factors and genetic determinants: the genetic background determines the ability to produce pigments, and environmental factors, such as light and temperature, precisely regulate the levels of anthocyanin accumulation. How these environmental factors interact with genetic factors to regulate species/cultivar-specific anthocyanin accumulation needs further study.
